# Genetic susceptibility to airway inflammation and exposure to short-term outdoor air pollution

**DOI:** 10.1186/s12940-023-00996-7

**Published:** 2023-06-29

**Authors:** Femke Bouma, Fredrik Nyberg, Anna-Carin Olin, Hanne Krage Carlsen

**Affiliations:** 1 Department of Occupational and Environmental Health, School of Public Health and Community Medicine, Institute of Medicine, Sahlgrenska Academy, University of Gothenburg, Medicinaregatan 16A, BOX 414, 40530 Gothenburg, Sweden; 2 School of Public Health and Community Medicine, Institute of Medicine, Sahlgrenska Academy, University of Gothenburg University, Gothenburg, Sweden

**Keywords:** Gene-environment interaction, Short-term air pollution exposure, Airway inflammation, FeNO, Quantile regression

## Abstract

**Background:**

Air pollution is a large environmental health hazard whose exposure and health effects are unequally distributed among individuals. This is, at least in part, due to gene-environment interactions, but few studies exist. Thus, the current study aimed to explore genetic susceptibility to airway inflammation from short-term air pollution exposure through mechanisms of gene-environment interaction involving the *SFTPA*, *GST* and *NOS* genes.

**Methods:**

Five thousand seven hundred two adults were included. The outcome measure was fraction of exhaled nitric oxide (FeNO), at 50 and 270 ml/s. Exposures were ozone (O_3_), particulate matter < 10 µm (PM_10_), and nitrogen dioxide (NO_2_) 3, 24, or 120-h prior to FeNO measurement. In the *SFTPA*, *GST* and *NOS* genes, 24 single nucleotide polymorphisms (SNPs) were analyzed for interaction effects. The data were analyzed using quantile regression in both single-and multipollutant models.

**Results:**

Significant interactions between SNPs and air pollution were found for six SNPs (*p* < 0.05): rs4253527 (*SFTPA1*) with O_3_ and NO_x_, rs2266637 (*GSTT1*) with NO_2_, rs4795051 (*NOS2*) with PM_10_, NO_2_ and NO_x_, rs4796017 (*NOS2*) with PM_10_, rs2248814 (*NOS2*) with PM_10_ and rs7830 (*NOS3*) with NO_2_. The marginal effects on FeNO for three of these SNPs were significant (per increase of 10 µg/m^3^):rs4253527 (*SFTPA1*) with O_3_ (β: 0.155, 95%CI: 0.013–0.297), rs4795051 (*NOS2*) with PM_10_ (β: 0.073, 95%CI: 0.00–0.147 (single pollutant), β: 0.081, 95%CI: 0.004–0.159 (multipollutant)) and NO_2_ (β: -0.084, 95%CI: -0.147; -0.020 (3 h), β: -0.188, 95%CI: -0.359; -0.018 (120 h)) and rs4796017 (*NOS2*) with PM_10_ (β: 0.396, 95%CI: 0.003–0.790).

**Conclusions:**

Increased inflammatory response from air pollution exposure was observed among subjects with polymorphisms in SFTPA1, GSTT1, and NOS genes, where O_3_ interacted with SFTPA1 and PM10 and NO_2_/NO_x_ with the GSTT1 and NOS genes. This provides a basis for the further exploration of biological mechanisms as well as the identification of individuals susceptible to the effects of outdoor air pollution.

**Supplementary Information:**

The online version contains supplementary material available at 10.1186/s12940-023-00996-7.

## Introduction

Adverse respiratory health effects due to air pollution are extensively researched, but the specific mechanisms through which air pollution affects different aspects of respiratory health are not completely understood [1]. However, the interplay between genetic and environmental factors is important in inducing asthma and other respiratory diseases [2].

Airway inflammation occurs when infectious or non-infectious agents infiltrate the lung tissue and trigger an inflammatory response. Fraction of exhaled nitric oxide (FeNO) is a non-invasive biomarker for airway inflammation [3, 4] which is elevated following short-term air pollution exposure in the general healthy population [5, 6], healthy children [7, 8], as well as adults with asthma [9], asthma and/or atopy [10], or in asthmatic children [11, 12]. The inter-individual variability in the inflammatory response characterized by FeNO may be explained by genetic variability [13].

Previously identified candidate genes, of which the protein products are thought to influence the molecular mechanism that underlie the effects of air pollution exposure, often form the basis for gene-environment interaction research. The amount or function of the protein product of a gene might be influenced by relevant polymorphisms in that gene, which can clarify mechanisms of susceptibility to air pollution-related health effects [14]. The genes encoding surfactant protein A (SP-A), Glutathione S-Transferase (GST) and Nitric Oxide Synthase (NOS) play potential roles in the association between short-term air pollution exposure and airway inflammation.

SP-A, encoded by two *STFPA* genes (*SFTPA1* and *SFTPA2),* forms an important component of pulmonary surfactant, a lipoprotein complex that consists of approximately 90% lipids and 10% proteins, including surfactant proteins A, B, C and D, with SP-A as the most abundant [15]. SP-A and SP-D play important roles in the host defense of the lungs as components of the innate and adaptive immune systems, interacting either directly with the pathogens or indirectly by activating immune cells and alleviating infection and inflammation in the lungs [16]. *SFTPA* gene variation is associated with acute and chronic lung diseases, both infectious and non-infectious, including asthma, COPD and lung cancer, as *SFTPA* genes are involved in inflammation mechanism [15]. Oxidation, a process induced by e.g. O_3_ exposure, modifies SP-A’s ability to enhance phagocytosis and stimulate cytokine production, two important functions of host defense [17, 18]. A lack of, or dysfunctional, SP-A might lead to increased oxidative stress and thus airway inflammation and increased FeNO [19].

*GST* genes encode proteins that are involved in the glutathione metabolism pathway, a part of the oxidative stress metabolism pathway. These enzymes detoxify endogenous and exogenous agents. Polymorphisms in these genes affect the functional oxidative capacity in the lungs and increase susceptibility to oxidative stress and thus airway inflammation [20]. The *GST* theta 1 (*GSTT1*) and *GST* pi 1 (*GSTP1*) genes are involved in detoxification of air pollutants and some polymorphisms are associated with increased susceptibility to air pollutants and other environmental toxicants [20]. They are involved in the anti-oxidant response and can increase or decrease the risk of adverse respiratory health outcomes [14], with heterogeneous results [21].

*NOS* gene SNPs and haplotype variations are associated with FeNO levels [22, 23]. The association between *NOS* and FeNO is modified by exposure to PM air pollution [8, 24]. Together, these findings suggest that gene-environment interaction could play a role with regards to air pollutants and the *NOS* genes.

Although genetic susceptibility seems to play an important role in the health effects experienced from air pollution exposure, evidence of its effects on population health is lacking as there are few studies investigating this in large cohorts. The aim of this study was to explore gene-environment interaction between *SFTPA*, *GST* and *NOS* SNPs and short-term exposure to air pollution on FeNO.

## Methods

### Study population

The study population consists of participants from the ADONIX (adult-onset asthma and exhaled NO) cohort of 6,686 men and women between the ages of 25 to 74 from the general population living in Gothenburg, Sweden [5]. The participants of this cohort were genotyped as part of the larger international INTERGENE-ADONIX cohort. Cross-sectional data consisting of clinical examinations and questionnaire answers were collected from 2001 until 2008 [25]. Current smokers (*n* = 984) were excluded from the analysis because smoking significantly influences the production of NO [26, 27], resulting in a study population of 5,702 participants.

### Exposure

Air pollution measurements were provided by the local environmental agency in Gothenburg (https://goteborg.se/wps/portal?uri=gbglnk%3a20120702-161602) as hourly mean concentrations from a roof-level monitoring station in the center of the city. To assess individual exposure for each study participant, the prior 3-, 24- and 120- hour averages of O_3_, PM_10_, NO_x_, and NO_2_ were matched to the time of clinical examination using the data presented in Modig et al. 2014 [5].

### Outcome

The outcome measure for airway inflammation was the biomarker FeNO, measured at the clinical examination using an online NO monitoring system at exhalation flow rates of 50 ml/s and 270 ml/s [25]. Lower flow rates, 50 ml/s (FeNO50), represent more proximal, bronchial, parts of the airways. Higher flow rates, at 270 ml/s (FeNO270), represent more distal, alveolar parts [28]. FeNO levels are highly influenced by age, height and atopy [29] and predicted values have been established [26].

### Genes

The selection of genes and SNPs for genotyping was based on previous literature on candidate genes,the theoretical background for potential interaction with air pollution and association with FeNO, as well as cost–benefit balance for the study. Furthermore, a focused a priori selection of genes decreases issues pertaining to multiple testing.

For GSTP1, GSTT1, SFTPA1, and SFTPA2 the genotyping was focused on a few specific candidate SNPs with prior association evidence. For GSTP1 eight SNPS were genotyped and four had reasonable coverage and were used in the analysis. One GSTT1 SNP was genotyped and was analysed. Four SFTPA1 SNPs were genotyped and used, and three SFTPA2 SNPs were genotyped successfully and used (one assay failed). These SNPs are the same as are previously reported from the same cohort [30]. For NOS2 and NOS3 we did broader tagging SNP genotyping (including also obviously known candidate SNPs) across the genes to capture LD and haplotype variation.

For NOS2 good coverage of 10 SNPs with low inter-SNP linkage disequilibrium (LD) based on haplotype analysis was observed in previous papers [22] and one more SNP was associated with FeNO was genotyped [23]. For NOS3 we used the SNP that was significantly related to FENO values in the 2012 paper [23].

Thus, in the current paper, a total of 24 SNPs in the *SFTPA*, *GST* and *NOS* genes were analysed.

For *SFTPA1* four SNPs were included, three for *SFTPA2*, four for *GSTP1*, one for *GSTT1*, 11 for *NOS2* and one for *NOS3*.

When investigating genetic association with certain disease traits, it is important to take different types of genetic modelling into account. SNPs are made up of major (A) and minor (a) alleles, whereby the minor allele represents the one that is less frequently present in the population. These alleles can occur in individuals in three different genotypes: homozygotic for the major allele (AA), heterozygotic (Aa) and homozygotic for the minor allele (aa). Some SNPs only occur with homozygotic genotypes. Commonly used models are the additive, dominant and recessive models [31]. Additive models compare the association between predictor and outcome variables across all different genotypes. Dominant models compare AA versus Aa and aa, recessive models compare aa versus AA and Aa [32]. The choice of genetic model in this study was based on the Kruskal–Wallis test of difference in median FeNO values across the genotype variations. If there was a significant difference (*p* < 0.05) between all three genotypes, an additive model was applied. In all other cases, a dominant model for the rare allele (based on homozygote major versus minor allele genotype frequencies) was applied.

### Covariates

Potential covariates were age, height, atopy, year and month of measurement, and current cold symptoms, and ambient temperature when available for the relevant exposure window. The inclusion of these covariates was based on an earlier study on air pollution and FeNO in this cohort [5]. Atopy was defined as having a positive Phadiatop IgE test (> 0.35 kUA/L) [33] and current cold was defined as having a cold or sore throat at the time of FeNO measurement, as self-reported from the questionnaire. Outside temperature was also included as a covariate in the analysis of air pollutants at 24 and 120 h, these data were not available for measurements at the 3 h exposure window.

### Statistical analysis

Descriptive statistics were performed for demographic characteristics of the study population, the distribution of FeNO, the allele frequencies of the SNPs and the estimates of air pollution exposure. Quantile regression was used to first investigate the association between short-term air pollution exposure and FeNO, because the distribution of both FeNO50 and FeNO270 were skewed. In quantile regression, the exposure is regressed on one or more percentiles of the outcome variable, and thus, 1) gives the possibility to investigate effects on the outcome variable beyond effects on the mean, and 2) does not rely on the assumption of normal distribution of the outcome variables [34]. The interpretation of the quantile regression coefficients is similar to those of linear regression, but instead of interpreting the parameter coefficients as the associations between a unit change in the exposure and the outcome variable *mean* value, the coefficient corresponds to a change in the outcome variable at the indicated percentile [35]. Examining associations between exposure and FeNO outside the mean value can give a more detailed picture of the effects, and previous studies have found stronger effects of genetics at the 50^th^ and 75^th^ percentiles of FeNO [36] and thus, we chose those percentiles to study associations beyond the mean, while maintaining adequate statistical power.

SNPs were added to the regression as interaction terms where both the main effects of the pollutant and the SNP and their interaction were included in the models. Interaction was tested in both single pollutant models as well as multipollutant models, where the main pollutant was added as an interaction term while the other pollutants were included in the model as covariates. Interaction terms with a *p*-value below 0.05 in the 50^th^ and/or 75^th^ percentile of FeNO were considered significant. When a significant interaction was found, the marginal effects, that is, the stratum-specific effects, of the air pollutant on FeNO across the different genotypes were calculated to clarify the direction and strength of the association [37]. The marginal effect coefficients per 10 µg/m^3^ increase in exposure are presented with their 95% confidence intervals (95% CI) and *p*-value for the interaction in the tables.

All statistical analyses were performed using StataSE 16. The study protocol for ADONIX was approved by the regional ethical review board.

## Results

### Descriptive results

Of all 5,702 participants, 3,819 had data on all relevant covariates and constitute the analysis population (Table 1). Of them, 3,780 had measured values of FeNO50, and 3,546 of FeNO270. The proportions of females and males were nearly equal, and the mean age was 51.4 years. Since current smokers were not included, 1,708 were former smokers and 2,111 had never smoked. The 50th and 75th percentile values of FeNO50 were 17.48 and 24.18 ppb, respectively. For FeNO270 these values were 5.55 and 7.26 ppb. FeNO50 and FeNO270 were significantly positively correlated (r spearman = 0.84). Air pollution exposure estimates were moderately high, with shorter exposure windows showing a larger range across the population (Table 2), and the pollutants were significantly correlated (Table A1). All genotype frequencies are shown in appendix Table A2.

**Table 1:** Characteristics of the study population (*n* = 3,819)

	**N (%)**
**Sex**	
Female; n (%)	1,947 (51.0%)
Male; n (%)	1,872 (49.0%)
**Age (years); mean ± SD**	51.4 (11.3)
**Height (centimeters); mean ± SD**	172.9 (9.2)
**Smoking**	
Never; n (%)	2,111 (55.3%)
Former; n (%)	1,708 (44.7%)
**Asthma; n (%)**	253 (6.7%)
**Atopy; n (%)**	936 (24.5%)
**Current cold; n (%)**	357 (9.4%)
**FeNO50 (ppb) (*n* = 3,780)**	
25^th^ percentile	12.9
50^th^ percentile	17.5
75^th^ percentile	24.2
**FeNO270 (ppb) (*n* = 3,546)**	
25^th^ percentile	4.2
50^th^ percentile	5.6
75^th^ percentile	7.26

**Table 2:** Air pollution exposure averaged over 3, 24 and 120 h before FeNO measurement, based on measurements from a central monitoring station 2001–2008. Distribution of exposures estimated in the study population (mean, quantiles). All units µg/m^3^

Air pollutant	Exposure window (hours)	Minimum	25^th^ %	Median	75^th^ %	Maximum	Mean (SD)
**O**_**3**_	3	0.1	22.6	44.4	61.8	126.8	43.4 (25.2)
	24	1.8	37.8	52.2	66.1	128.3	51.4 (20.6)
	120	6.7	41.6	53.0	63.7	107.2	52.5 (15.9)
**PM**_**10**_	3	0.3	16.6	23.3	32.5	171.0	27.1 (16.4)
	24	4.9	16.8	21.7	28.1	76.0	23.8 (10.3)
	120	9.5	18.1	21.9	26.8	64.6	23.4 (8.1)
**NO**_**2**_	3	5.7	19.0	28.0	42.0	250.3	34.1 (22.4)
	24	5.1	17.1	23.5	32.3	104.9	26.5 (12.9)
	120	10.5	18.6	22.9	29.2	71.7	24.8 (8.9)
**NO**_**x**_	3	5.7	22.8	35.7	62.8	981.0	62.3 (78.6)
	24	6.0	19.8	28.8	48.6	330.9	40.6 (34.0)
	120	12.0	23.0	30.2	43.1	317.1	36.9 (23.8)

### Air pollution and FENO

In adjusted single-pollutant models, only 3 h- and 120 h-average O_3_ were significantly associated, with FeNO270 at the 50^th^ percentile. Although few coefficients are statistically significant, the results are fairly consistent across the quantiles (Table 3, unadjusted results in Table A3).

**Table 3:** Adjusted associations between FeNO and air pollutants PM_10_, NO_2_, NOx, and O_3_ estimated from quantile regression

	**FeNO50 (ppb)**^**a**^ **β coefficient (95% CI)**		**FeNO270 (ppb)**^**b**^ **β coefficient (95% CI)**	
**O**_**3**_	50th %	75th %	50th %	75th %
3 h	0.105 (-0.046, 0.255)	0.053 (-0.201, 0.307)	**0.040 (0.002, 0.078)**	0.023 (-0.036, 0.082)
24 hrs^c^	-0.047 (-0.268, 0.174)	-0.106 (-0.494, 0.282)	0.031 (-0.030, 0.091)	-0.023 (-0.113, 0.068)
120 hrs^c^	0.193 (-0.151, 0.537)	0.122 (-0.487, 0.731)	**0.098 (0.007, 0.188)**	0.081 (-0.059, 0.221)
**PM**_**10**_				
3 h	-0.068 (-0.278, 0.142)	-0.233 (-0.585, 0.120)	0.016 (-0.038, 0.071)	0.005 (-0.078, 0.087)
24 hrs^c^	-0.222 (-0.554, 0.111)	-0.396 (-0.994, 0.201)	0.024 (-0.067, 0.114)	-0.030 (-0.166, 0.106)
120 hrs^c^	-0.183 (-0.619, 0.254)	-0.373 (-1.183, 0.438)	0.042 (-0.077, 0.160)	0.030 (-0.153, 0.213)
**NO**_**2**_				
3 h	-0.058 (-0.211, 0.095)	-0.163 (-0.419, 0.093)	-0.024 (-0.064, 0.015)	-0.028 (-0.085, 0.029)
24 hrs^c^	-0.028 (-0.305, 0.250)	0.033 (-0.457, 0.523)	0.016 (-0.060, 0.091)	0.055 (-0.058, 0.167)
120 hrs^c^	-0.280 (-0.684, 0.124)	-0.384 (-1.111, 0.345)	-0.019 (-0.129, 0.090)	-0.030 (-0.193, 0.132)
**NO**_**x**_				
3 h	-0.011 (-0.054, 0.032)	-0.042 (-0.115, 0.032)	-0.001 (-0.012, 0.011)	-0.003 (-0.019, 0.013)
24 hrs^c^	-0.021 (-0.129, 0.088)	0.033 (-0.157, 0.224)	0.009 (-0.020, 0.038)	0.007 (-0.037, 0.051)
120 hrs^c^	-0.112 (-0.272, 0.048)	-0.213 (-0.498, 0.072)	-0.0004 (-0.043, 0.042)	-0.025 (-0.091, 0.040)

All models are adjusted for age, height, year, month, current cold, and atopy. All estimates are given per 10 µg/m^3^ increase in air pollutant exposure

^a^*n* = 3780 for 3 h, *n* = 3770 for 24 and 120 h

^b^*n* = 3546 for 3 h, *n* = 3536 for 24 and 120 h

^c^Also adjusted for temperature

### Gene-environment interaction analysis

Analyzing the effects of gene-environment interactions on FeNO, we observed significant interactions (*p* < 0.05) in the 50^th^ and/or 75^th^ percentile of FeNO for six SNPs: rs4253527 *SFTPA1*), rs2266637 (*GSTT1*), rs4795051 (*NOS2*), rs4796017 (*NOS2*), rs2248814 (*NOS2*) and rs7830 (*NOS3*). The results for FeNO50 at the 50^th^ percentile are shown in Table 4. Table 5 contains the results for FeNO270 at the 50^th^ percentile and Table 6 the results for FeNO270 at the 75^th^ percentile.

**Table 4:** Gene-environment interactions between pollutant exposure and genotypes on FeNO50 estimated from single and multipollutant quantile regression models (50^th^ percentile)

SNP	Pollutant (exposure window)	Single pollutant				Multi pollutant			
		*p*-value^a^	Marginal effect coefficient (95%CI)			*p*-value^a^	Marginal effect coefficient (95%CI)		
**rs7830**^**b**^** (NOS3) (*n* = 3599)**	NO_2_ (24 h)	0.047	**GG** 0.259 (-0.150, 0.668)	**TG/TT** -0.264 (-0.628, 0.100)		0.033	**GG** 0.409 (-0.277, 1.094)	**TG/TT** -0.149 (-0.825, 0.526)	
**rs4795051**^**c**^** (NOS2) (*n* = 3612)**	NO_x_ (3 h)	0.155 (GC)	**CC** -0.060 (-0.137, 0.016)	**GC** 0.012 (-0.053, 0.076)	**GG** -0.033 (-0.145, 0.080)	0.048 (GC)	**CC** -0.027 (-0.153, 0.098)	**GC** 0.073 (-0.043, 0.189)	**GG** 0.034 (-0.111, 0.179)

All 3-h interaction analyses are adjusted for age, height, year, month, current cold, and atopy. All 24- and 120-h interaction analyses are adjusted for age, height, year, month, current cold, atopy, and temperature. All estimates are given per 10 µg/m^3^ increase in air pollutant exposure

^a^*p*-value for interaction term. For additive models the genotype with significant difference from the major allele genotype is presented in parentheses

^b^Dominant model

^c^Additive model

**Table 5:** Gene-environment interactions between pollutant exposure and genotypes on FeNO270 estimated from single and multipollutant quantile regression models (50^th^ percentile)

SNP	Pollutant (exposure window)	Single pollutant				Multi pollutant			
		*p*-value^a^	Marginal effect coefficient (95%CI)			*p*-value^a^	Marginal effect coefficient (95%CI)		
**rs2266637 (*GSTT1*) (*n* = 2880)**	NO_2_ (120 h)	0.038	**CC** -0.113 (-0.255, 0.030)	**GG** 0.170 (-0.063, 0.403)		0.019	**CC** -0.035 (-0.314, 0.244)	**GG** 0.298 (-0.080, 0.677)	
**rs4795051**^**b**^** (*NOS2*) (*n* = 3398)**	PM_10_ (3 h)	0.003 (GC)	**CC** -0.092 (-0.179, -0.006)	**GC** 0.073 (0.000, 0.147)	**GG** 0.007 (-0.107, 0.120)	0.004 (GC)	**CC** -0.079 (-0.169, 0.011)	**GC** 0.081 (0.004, 0.159)	**GG** 0.028 (-0.088, 0.143)
	PM_10_ (24 h)	0.050 (GC)	**CC** -0.111 (-0.262, 0.040)	**GC** 0.075 (-0.044, 0.195)	**GG** 0.018 (-0.190, 0.195)	0.066 (GC)	**CC** -0.129 (-0.287, 0.029)	**GC** 0.045 (-0.085, 0.175)	**GG** -0.004 (-0.218, 0.210)
	NO_2_ (24 h)	0.039 (GG)	**CC** -0.051 (-0.173, 0.072)	**GC** -0.009 (-0.106, 0.088)	**GG** 0.163 (-0.007, 0.333)	0.036 (GG)	**CC** -0.059 (-0.241, 0.124)	**GC** -0.011 (-0.184, 0.162)	**GG** 0.157 (-0.075, 0.389)
	NO_2_ (120 h)	0.004 (GG)	**CC** -0.188 (-0.359, -0.018)	**GC** -0.007 (-0.150, 0.136)	**GG** 0.234 (-0.008, 0.475)	0.007 (GG)	**CC** -0.213 (-0.507, 0.082)	**GC** -0.049 (-0.308, 0.209)	**GG** 0.189 (-0.146, 0.524)
	NO_x_ (120 h)	0.017 (GG)	**CC** -0.057 (-0.115, 0.002)	**GC** 0.005 (-0.055, 0.064)	**GG** 0.065 (-0.022, 0.152)	0.035 (GG)	**CC** -0.014 (-0.106, 0.078)	**GC** 0.051 (-0.054, 0.155)	**GG** 0.094 (-0.022, 0.209)
**rs2248814**^**b**^** (*NOS2*)** **(*n* = 3398)**	PM_10_ (3 h)	0.033 (GA) 0.046 (AA)	**GG** -0.070 (-0.157, 0.017)	**GA** 0.055 (-0.023, 0.132)	**AA** 0.078 (-0.041, 0.197)	0.042 (GA) 0.079 (AA)	**GG** -0.063 (-0.153, 0.027)	**GA** 0.052 (-0.028, 0.133)	**AA** 0.064 (-0.056, 0.184)

All 3-h interaction analyses are adjusted for age, height, year, month, current cold, and atopy. All 24- and 120-h interaction analyses are adjusted for age, height, year, month, current cold, atopy, and temperature. All estimates are given per 10 µg/m^3^ increase in air pollutant exposure

^a^*p*-value for interaction term. For additive models the genotype with significant difference from the major allele genotype is presented in parentheses

^b^Additive model

**Table 6:** Gene-environment interactions between pollutant exposure and genotypes on FeNO270 estimated from single and multipollutant quantile regression models (75^th^ percentile)

SNP	Pollutant (exposure window)	Single pollutant				Multi pollutant			
		*p*-value^a^	Marginal effect coefficient (95%CI)			*p*-value^a^	Marginal effect coefficient (95%CI)		
**rs4253527**^**b**^** (SFTPA1) (*n* = 3052)**	O_3_ (3 h)	0.029	**CC** -0.015 (-0.088, 0.058)	**TC/TT** 0.155 (0.013, 0.297)		0.019	**CC** -0.028 (-0.125, 0.069)		**TC/TT** 0.142 (-0.014, 0.299)
	NO_x_ (3 h)	0.050	**CC** 0.009 (-0.012, 0.029)	**TC/TT** -0.0371 (-0.078, 0.004)		0.089	**CC** 0.027 (-0.018, 0.072)		**TC/TT** -0.014 (-0.075, 0.046)
**rs4796017**^**c**^** (NOS2) (*n* = 3378)**	PM_10_ (120 h)	0.047 (GG)	**AA** -0.091 (-0.394, 0.212)	**GA** -0.125 (-0.371, 0.121)	**GG** 0.396 (0.003, 0.790)	0.077 (GG)	**AA** -0.032 (-0.362, 0.297)	**GA** -0.040 (-0.318, 0.238)	**GG** 0.404 (-0.011, 0.819)

All 3-h interaction analyses are adjusted for age, height, year, month, current cold, and atopy. All 24- and 120-h interaction analyses are adjusted for age, height, year, month, current cold, atopy, and temperature. All estimates are given per 10 µg/m^3^ increase in air pollutant exposure

^a^*p*-value for interaction term. For additive models the genotype with significant difference from the major allele genotype is presented in parentheses

^b^Dominant model

^c^Additive model

### FeNO50 at the 50^th^ percentile

For FeNO50 at the 50^th^ percentile (Table 4), rs7830 (*NOS3*) showed significant interactions with 24 h-average NO_2_ in both the single- and multipollutant models. Rs4795051 (*NOS2*) had significant interaction with 3 h-average NO_x_ for FeNO50 at the 50^th^ percentile, in the multipollutant model, the single pollutant model showed similar patterns, but did not reach significance (Table 4, all exposure windows are shown in Table A4).

### FeNO270 at the 50^th^ percentile

For FeNO270 at the 50^th^ percentile, the *GSTT1* SNP rs2266637 had a significant interaction with 120 h-average NO_2_ in both single- and multipollutant models (Table 5, all exposure windows are shown in Table A5).

For FeNO270 at the 50^th^ percentile, the *NOS2* SNP rs4795051 had significant interactions with 24 h- and 120 h-average NO_2_, 120 h-average NO_x_ and 3 h- and 24 h-average PM_10_, however, the results only reached statistical significance in the single pollutant model for 24 h-average PM_10_ although the estimated coefficients were very similar. Another *NOS2* SNP, rs2248814, interacted with 3 h-average PM_10_ on FeNO270 in both single- and multipollutant models (Table 5, all exposure windows are shown in Table A6).

### FeNO270 at the 75^th^ percentile

One SNP on the *SFTPA1* gene, rs4253527, interacted significantly with exposure to 3 h-average O_3_ in both the single and multipollutant models for FeNO270 at the 75^th^ percentile (Table 6, all exposure windows are shown in Table A7). This SNP also had a significant interaction with 3 h-average NO_x_ in the single pollutant model, but with opposite directions of the marginal effects (Table 6, all exposure windows are shown in Table A7). Lastly, analyzing FeNO270 at the 75^th^ percentile (Table 6), the *NOS2* SNP rs4796017 interacted significantly with 120 h-average PM_10_ in the single pollutant model (Table 6, all exposure windows are shown in Table A6).

The interactions of SNPs on the associations between specific air pollutants and FeNO, are illustrated for SNPs with significant interactions in the appendix (Figs. A1, A2, A3, A4, A5, A6, A7 and A8) for predicted values of FeNO for the 25^th^ to the 75^th^ percentile values of the relevant air pollutants.

## Discussion

In our study, we observed suggested signs of gene-environment interaction of short-term air pollution exposure with SNPs from each of the investigated candidate gene categories of *SFTPA*, *GST* and *NOS* on FeNO. Of the 24 SNPs analyzed in this study, six had significant interactions with at least one air pollutant in at least one of the included exposure windows on FeNO at different percentiles. We noted that O_3_ mainly interacted with *SFTPA1*, whereas PM_2.5_ and NO_2_/NO_x_ had significant interactions with *GSTT1, NOS2 and NOS3.*

The direction of the marginal effect associations differed between the different genes. For *NOS2* and *GSTT1* SNPs with significant air pollution interactions, the marginal effects indicated a positive association between air pollution exposure and FeNO in minor allele or heterozygote genotypes compared with the major allele genotypes. This was the opposite for *NOS3*, where the marginal effects indicated associations between air pollution exposure and increased FeNO for the major allele carriers. In *SFTPA*1, opposing directions of association were observed. O_3_ exposure was associated with increased FeNO in minor allele carriers, whereas NO_x_ exposure was associated with increased FeNO in homozygote major allele carriers (Table 6). The opposing direction of marginal effects associated with NO_x_ and O_3_ are likely related to their negative correlations (Appendix Table A1), which also complicates any interpretation of this result.

### *NOS* genes

The *NOS3* polymorphism rs7830 interacted significantly with 24 h-average exposure to NO_2_ on FeNO50 at the 50^th^ percentile with the minor allele genotype displaying a negative slope (Table 4). This is in line with what was previously reported for nicotine exposure and FeNO, where higher nicotine exposure was associated with lower FeNO levels in individuals with rs7830 TG/TT genotypes [38]. A study of the effects of *NOS3* polymorphisms on the association between short-term PM_10_ exposure and oxidative stress markers found that some haplotypes which included rs7830 were at risk for higher levels of oxidative stress [39]. As oxidative stress can be a cause of airway inflammation, this suggests a possible mechanism for the interaction between air pollutants and *NOS3* polymorphisms on FeNO by. Although these studies did not specifically investigate exposure to NO_2_, they indicate that the effects of pollutants and oxidants, including NO_2_, can be modified by *NOS3* and specifically the rs7830 SNP (Table 4).

The *NOS2* SNP rs4795051 exhibited significant interactions for the heterozygote genotype (GC) with PM_10_, and for the minor allele genotype (GG) with NO_2_ and NOx (Table 5), both with positive marginal associations with FeNO270 at the 50^th^ percentile. Similar effect directions were seen for the *NOS2* SNPs rs2248814 (Table 5) and rs4796017 (Table 6) with PM_10_ on FeNO270 at the 75^th^ percentile, where the heterozygote genotypes and/or the minor allele genotypes were positive associated with FeNO compared to the major allele genotypes. *NOS2* encodes the enzyme iNOS, which is the main determinant of FeNO and is induced by different stimuli. An increase in iNOS response, which might be present in the heterozygote and/or minor allele genotypes, could mean an increase in effective inflammatory response, thereby protecting the lungs from pathogens and other stressors. On the contrary, it might mean these genotypes have an adverse effect on pulmonary health as they entail an increase in inflammation, and inflammation can itself be damaging to the lungs.

Short-term exposure to NO_2_, PM_2.5_, a constituent of PM_10_, and traffic-related air pollution have all been associated with lower *NOS2* methylation, leading to higher expression of iNOS and thus higher levels of FeNO [35]. Furthermore, individuals with different *NOS2* haplotypes were found to experience different degrees of methylation, pointing towards a combination of genetic and epigenetic effects [8]. In children, this was further investigated using quantile regression and the associations between PM_2.5_ exposure, genetic and epigenetic effects and FeNO were more prominent among those with higher FeNO levels [35]. Epigenetic variations, such as differences in methylation, among specific SNPs have not been reported, but they might play a role in the interactions that we found and could be a target for further research.

### *GST* genes

The minor allele genotype of the *GSTT1* SNP rs2266637 was also positively associated with FeNO270 at the 50^th^ percentile compared to its major allele genotype with exposure to 120 h-average NO_2_ exposure (Table 5). Given the anti-oxidant properties of GST [20], this also suggests a dysfunctional enzyme in individuals with the minor allele genotype, leading to higher levels of airway inflammation as oxidation due to NO_2_ exposure is less inhibited. Previous research into *GSTT1* and respiratory health effects has mainly focused on differences between null, i.e. homozygous deletion, and non-null genotypes. Results from some of these studies have supported the presence of interaction but others found none [20, 40]. Specifically with NO_2_ exposure or traffic-related air pollution, some found interaction with *GSTT1* [41–43] whereas others found nonE [44, 45]. Variations in study design, the outcome investigated or differences in allele frequencies based on ethnicity could be responsible for heterogeneity in the results [21].

### *SFTPA* gene

Our results showed that for *SFTPA*, the SNP rs4253527 on the *SFTPA1* gene had significant interaction with 3 h-average exposure to O_3_ where minor allele genotypes were positively associated with FeNO270 at the 75^th^ percentile (Table 6). *SFTPA* plays an important role in host defense of the lungs and aids in the alleviation of inflammation [16]. This SNP, rs4253527, is characteristic for the *SFTPA1* 6A4 haplotype as it changes the amino acid encoded by this haplotype [46]. This is thought to result in a change in surfactant protein structure and function, affecting the carrier’s disease susceptibility [47]. Oxidation of SP-A, an effect of O_3_ exposure, has been found to decrease the ability of SP-A to enhance phagocytic activity and cytokine production. The effects of oxidation differ between *SFTPA1* and *SFTPA2* haplotypes, with a seemingly stronger effect on *SFTPA2* [17]. Whether these effects and differences therein also occur between different SNPs in the respective genes have not been elucidated, but this may play a role in the differential FeNO levels among the SNP genotypes found in this study.

### FeNO and results related to exhalation flow

For FeNO50, only two SNPs showed significant interactions whereas more interactions were found for FeNO270, which represents NO produced in the more distal parts of the airways [28]. Previous studies have reported more influences of short-term air pollution exposure on FeNO at higher flow rates and postulate that air pollution-induced inflammation processes likely occur in the smaller, distal airways [5]. Therefore, our observation of more significant genetic interactions for FeNO270 – a distal airway biomarker—than for FeNO50, are plausible.

### Exposure windows

Currently, there is no consensus on which time lag, interval, or average of pollution exposure are most appropriate for observing effects of short-term air pollution exposure on FeNO, so the different interactions for different exposure windows for different pollutants observed in our study are difficult to interpret. The previous study within this cohort reported that the longer 120-h average seemed more applicable for O_3_ whereas the 24-h average seemed more applicable for NO_x_ [5]. In our study, which is based on a slightly different study population and methods, only O_3_ at 120 h and FeNO270, and NO_2_ at 120 h and FeNO50 had positive associations in either the crude (Table A2) or adjusted models (Table 4). The crude models showed additional associations with NO_2_ and NO_x_ at 24 and 120 h, and the adjusted models was additionally associated with O_3_ at 3 h.

Interestingly, introducing interaction terms increased the number of pollutants and exposure windows with significant associations with FeNO, such as PM_10_, and NO_x_ at 3 h, indicating that genetic predisposition and susceptibility are important when studying air pollution health effects. Also, the interactions occurred at different exposure windows for the different pollutants, although the results for different exposure windows tended to be similar, and in some cases had indications of a dose–response pattern (e.g. Table A5 rs2266637 gg allele). Whereas most pollutants had interactions at more than one exposure window, O_3_ only interacted at the 3-h exposure window, with *SFTPA* SNP rs4253527 indicating that the effect of O_3_ were short-acting and transient. PM_10_ had interactions at 3, 24, and 120 h average exposure with either of the two *NOS2* SNPs for FeNO270, which might be due to the compositional complexity of PM_10_ having multiple pathways for generating a FeNO response. All significant interactions for NO_2_ and NO_x_ on FeNO270 were at the 24- and 120-h exposure windows, indicating a longer time window of the FeNO response, and *SFTPA* interactions were only observed at 3 or 24 h average exposure to O_3_ and NO_2_, indicating a faster biological response.

However, since only a limited selection of exposure windows and genes were tested in this study, more research is needed to further clarify the time windows in which gene-environment interaction can occur.

### Quantiles

Our results were not homogenous across quantiles of FeNO, something that has previously been reported [35]. Zhang and colleagues found that joint effects of air pollution exposure and genetic and epigenetic variants occurred mainly in the upper tail of the FeNO distribution in children, suggesting that children with high FeNO have increased (epi)genetic susceptibility to air pollution, which supports what was observed in the current study in adults.

### Single and multipollutant models

Results from single-and multipollutant models were in most cases largely similar (exceptions are 3-h NO_x_ and 24-h PM_10_), indicating that the effects of individual pollutants are relatively independent of each other. For FeNO270, the rs4253527 interactions with NO_x_ and O_3_ with opposite directions in single-pollutant models, which remained only for O_3_ in the multipollutant model (Table 6), may be explained by the negative correlation between NO_x_ and O_3_ in our data and which is commonly seen (Table A1).

### Strengths and limitations

In this study, air pollution exposure was assigned based on measurements from centrally located air pollution monitors, which provides a rough estimate of the true exposure of individuals as the distances to the residences of the participants varied, although all participants lived in the city of Gothenburg [48]. On the other hand, all clinical data were collected at the same site, so for the 3-h average exposure in particular, all participants would have quite comparable accuracy of exposure. The data used for this study is relatively old, as it was collected between 2001 and 2008, which could affect the generalizability of the results to present day conditions of the same area, as air pollution exposure levels and composition have changed drastically over the past decades. However, the mechanism of genetic susceptibility to air pollution would not be expected to change over time and the pollutants that were investigated are still present in current urban air pollution. In our data, both NO_2_ and NO_x_ variables have a skewed distribution, but, short-term exposure to air pollution is extremely variable among individuals, because of its dependence on weather, spatiotemporal variability, time spent outside, and traffic. Therefore, the mean exposure was deemed to be more representative of the true exposure of individuals.

A limitation of all gene-environment interaction research that investigates certain SNPs or haplotypes is that only a selection of genetic information is included, which implies that even when no interactions are found, they might be present with other SNPs or haplotypes that were not included in the analysis. Another limitation is the lack of replication analysis, due to difficulty of finding a suitable replication cohort.

The number of hypotheses tested in gene-environment studies can lead to false positives and to ensure the validity of positive results, adjustment for multiple comparisons is sometimes advised, especially when testing is hypothesis-free. As this study aimed to analyse associations based on a-priori hypotheses based on plausible biological mechanisms and employed a selected number of SNPs, no adjustments for multiple comparisons [49, 50].

## Conclusion

Airway inflammation induced by short-term effects of air pollution exposure can accumulate over time and contribute to long-term symptoms, disease and even mortality. Genetic susceptibility to the effects of air pollution are associated with greater inflammatory response, putting individuals with certain genetic markers at greater risk. In this exploratory study, interactions between O_3_ and *SFTPA1,* and PM_10_ and NO_2_/NO_x_ with *GSTT1* and *NOS* genes were found to play a role in the association between short-term air pollution and FeNO, suggesting an increased inflammatory response among subjects with polymorphisms in these genes. These findings contribute to the understanding of the role that genetic susceptibility can play in the health effects of outdoor air pollution and provide a basis for the further exploration of biological mechanisms as well as the identification of susceptible populations and individuals.

## Supplementary Information

**Additional file 1: Table A1.** Pearson correlation coefficients for all air pollutants at 3-, 24- and 120-hour average concentrations based on the study population exposure estimates. **Table A2.** Genotype frequencies. **Table A3.** Unadjusted associations between FeNO and air pollutants PM_10_, NO_2_, NOx, and O_3_ estimated from quantile regression (crude models). **Table A4.** Gene-environment interactions between pollutant exposure and genotypes on FeNO50 estimated from single and multipollutant quantile regression models (50^th^ percentile). **Table A5.** Gene-environment interactions between pollutant exposure and genotypes on FeNO270 estimated from single and multipollutant quantile regression models (GST genes). **Table A6.** Gene-environment interactions between pollutant exposure and genotypes on FeNO270 estimated from single and multipollutant quantile regression models (NOS genes). **Table A7.** Gene-environment interactions between pollutant exposure and genotypes on FeNO270 estimated from single and multipollutant quantile regression models (SFTPA genes). **Figure A1.** SFTPA1 SNPs: Plots of predictive margins with significant interactions. (A) rs4253527 with O_3_ at 3 hours, single pollutant model. (B) rs4253527 with O_3_ at 3 hours, multi pollutant model. (C) rs4253527 with NOx at 3 hours, single pollutant model. **Figure A2.** GSTT1 SNPs: Plots of predictive margins with significant interactions. (A) rs2266637 with NO_2_ at 120 hours, single pollutan tmodel. (B) rs2266637 with NO_2_ at 120 hours, multi pollutant model. **Figure A3.** NOS2 SNPs: Plots of predictive margins with significant interactions. (A) rs4795051 with PM_10_ at 3 hours, single pollutant model. (B) rs4795051 with PM_10_ at 3 hours, multi pollutant model. (C) rs4795051 with PM_10_ at 24 hours, single pollutant model. **Figure A4.** NOS2 SNPs: Plots of predictive margins with significant interactions. (A) rs4795051 with NO_2_ at 24 hours, single pollutant model. (B) rs4795051 with NO_2_ at 24 hours, multi pollutant model. (C) rs4795051 with NO_2_ at 120 hours, single pollutant model. (D) rs4795051 with NO_2_ at 120 hours, multi pollutant model. **Figure A5.** NOS2 SNPs: Plots of predictive margins with significant interactions. (A) rs4795051 with NO_x_ at 3 hours, multi pollutant model. (C) rs4795051 with NO_x_ at 120 hours, single pollutant model. (D) rs4795051 with NO_x_ at 120 hours, multi pollutant model. **Figure A6.** NOS2 SNPs: Plots of predictive margins with significant interactions. rs4796017 with PM_10_ at 120 hours, single pollutant model. **Figure A7.** NOS2 SNPs: Plots of predictive margins with significant interactions. (A) rs2248814 with PM_10_ at 3 hours, single pollutant model. (B) rs2248814 with PM_10_ at 3 hours, multi pollutant model. **Figure A8.** NOS3 SNPs: Plots of predictive margins with significant interactions. (A) rs7830 with NO_2_ at 24 hours, single pollutant model. (B) rs7830 with NO_2_ at 24 hours, multi pollutant model.

## Data Availability

The data are available opon reasonable request and approval of science ethics review board.
