# Supplementary material for: Genetic susceptibility to airway inflammation and exposure to short-term outdoor air pollution
Source: Environ Health. 2023 Jun 29;22:50. doi: 10.1186/s12940-023-00996-7 (PMC10308777; doi:10.1186/s12940-023-00996-7)
Supplement: Supplementary file 1 — Additional file 1: Table A1. Pearson correlation coefficients for all air pollutants at 3-, 24- and 120-hour average concentrations based on the study population exposure estimates. Table A2. Genotype frequencies. Table A3. Unadjusted associations between FeNO and air pollutants PM10, NO2, NOx, and O3 estimated from quantile regression (crude models). Table A4. Gene-environment interactions between pollutant exposure and genotypes on FeNO50 estimated from single and multipollutant quantile regression models (50th percentile). Table A5. Gene-environment interactions between pollutant exposure and genotypes on FeNO270 estimated from single and multipollutant quantile regression models (GST genes). Table A6. Gene-environment interactions between pollutant exposure and genotypes on FeNO270 estimated from single and multipollutant quantile regression models (NOS genes). Table A7. Gene-environment interactions between pollutant exposure and genotypes on FeNO270 estimated from single and multipollutant quantile regression models (SFTPA genes). Figure A1. SFTPA1 SNPs: Plots of predictive margins with significant interactions. (A) rs4253527 with O3 at 3 hours, single pollutant model. (B) rs4253527 with O3 at 3 hours, multi pollutant model. (C) rs4253527 with NOx at 3 hours, single pollutant model. Figure A2. GSTT1 SNPs: Plots of predictive margins with significant interactions. (A) rs2266637 with NO2 at 120 hours, single pollutan tmodel. (B) rs2266637 with NO2 at 120 hours, multi pollutant model. Figure A3. NOS2 SNPs: Plots of predictive margins with significant interactions. (A) rs4795051 with PM10 at 3 hours, single pollutant model. (B) rs4795051 with PM10 at 3 hours, multi pollutant model. (C) rs4795051 with PM10 at 24 hours, single pollutant model. Figure A4. NOS2 SNPs: Plots of predictive margins with significant interactions. (A) rs4795051 with NO2 at 24 hours, single pollutant model. (B) rs4795051 with NO2 at 24 hours, multi pollutant model. (C) rs4795051 with NO2 at [file 12940_2023_996_MOESM1_ESM.docx]

# APPENDIX MATERIAL

#### Table A1. Pearson correlation coefficients for all air pollutants at 3-, 24- and 120-hour average concentrations based on the study population exposure estimates

| Pollutant |  | O_3_ | | | PM_10_ | | | NO_2_ | | | NO_x_ | | |
| --- | --- | --- | --- | --- | --- | --- | --- | --- | --- | --- | --- | --- | --- |
|  | Lag time | 3 | 24 | 120 | 3 | 24 | 120 | 3 | 24 | 120 | 3 | 24 | 120 |
| O_3_ | 3 | 1 |  |  |  |  |  |  |  |  |  |  |  |
|  | 24 | 0.72 | 1 |  |  |  |  |  |  |  |  |  |  |
|  | 120 | 0.57 | 0.78 | 1 |  |  |  |  |  |  |  |  |  |
| PM_10_ | 3 | -0.05 | 0.06 | 0.09 | 1 |  |  |  |  |  |  |  |  |
|  | 24 | 0.11 | 0.15 | 0.19 | 0.69 | 1 |  |  |  |  |  |  |  |
|  | 120 | 0.17 | 0.21 | 0.25 | 0.49 | 0.72 | 1 |  |  |  |  |  |  |
| NO_2_ | 3 | -0.64 | -0.36 | -0.23 | 0.39 | 0.17 | 0.09 | 1 |  |  |  |  |  |
|  | 24 | -0.4 | -0.55 | -0.28 | 0.32 | 0.32 | 0.19 | 0.65 | 1 |  |  |  |  |
|  | 120 | -0.23 | -0.31 | -0.39 | 0.27 | 0.26 | 0.36 | 0.45 | 0.66 | 1 |  |  |  |
| NO_x_ | 3 | -0.56 | -0.38 | -0.26 | 0.38 | 0.13 | 0.03 | 0.91 | 0.60 | 0.4 | 1 |  |  |
|  | 24 | -0.42 | -0.56 | -0.36 | 0.25 | 0.23 | 0.06 | 0.62 | 0.89 | 0.56 | 0.68 | 1 |  |
|  | 120 | -0.3 | -0.4 | -0.51 | 0.17 | 0.14 | 0.19 | 0.42 | 0.6 | 0.88 | 0.43 | 0.62 | 1 |

Note: All coefficients had p-values < 0.05

#### Table A2. Genotype frequencies

|  | Major allele homozygote  Genotype n (%) | Heterozygote  Genotype n (%) | Minor allele homozygote  Genotype n (%) |
| --- | --- | --- | --- |
| SP-A1 |  |  |  |
| rs1136450 | GG 1845 (36.8%) | GC 3152 (62.9%) | CC 11 (0.2%) |
| rs1136451 | AA 3522 (71.0%) | GA 1314 (26.5%) | GG 122 (2.5%) |
| rs1059057 | AA 4413 (87.8%) | GA 593 (11.8%) | GG 20 (0.4%) |
| rs4253527 | CC 4120 (82.1%) | TC 852 (17.0%) | TT 45 (0.9%) |
| SP-A2 |  |  |  |
| rs1059046 | TT 1925 (38.6%) | TG 2369 (47.5%) | GG 692 (13.9%) |
| rs1965707 | GG 2689 (54.1%) | GA 1959 (39.4%) | AA 327 (6.6%) |
| rs1965708 | GG 3318 (66.1%) | TG 1535 (30.6%) | TT 169 (3.4%) |
| GSTP1 |  |  |  |
| rs1695 | AA 2427 (45.4%) | GA 2330 (43.6%) | GG 585 (11.0%) |
| rs1138272 | CC 4610 (85.7%) | TC 734 (13.6%) | TT 33 (0.6%) |
| rs762803 | CC 1774 (33.2%) | CA 2597 (48.6%) | AA 974 (18.2%) |
| rs596603 | GG 1730 (32.3%) | TG 2622 (48.9%) | TT 1011 (18.9%) |
| GSTT1 |  |  |  |
| rs2266637 | CC 3157 (69.7%) | - | GG 1371 (30.3%) |
| NOS2 |  |  |  |
| rs2248814 | GG 1909 (35.6%) | GA 2577 (48.0%) | AA 880 (16.4%) |
| rs2779248 | TT 2023 (37.8%) | CT 2534 (47.3%) | CC 799 (14.9%) |
| rs10459953 | GG 2222 (41.5%) | GC 2437 (45.6%) | CC 691 (12.9%) |
| rs12944039 | GG 3322 (62.0%) | GA 1802 (33.6%) | AA 238 (4.4%) |
| rs2297514 | TT 1904 (35.6%) | CT 2545 (47.6%) | CC 893 (16.7%) |
| rs9901734 | CC 3151 (58.6%) | GC 1924 (35.8%) | GG 299 (5.6%) |
| rs4795051 | CC 1755 (32.7%) | GC 2736 (51.0%) | GG 873 (16.3%) |
| rs4796017 | AA 1749 (32.8%) | GA 2564 (48.0%) | GG 1026 (19.2%) |
| rs2297520 | CC 1900 (35.6%) | TC 2577 (48.3%) | TT 856 (16.1%) |
| rs9895453 | TT 1460 (27.3%) | CT 2681 (50.1%) | CC 1213 (22.7%) |
| rs3729508 | CC 1915 (35.7%) | TC 2590 (48.1%) | TT 860 (16.0%) |
| NOS3 |  |  |  |
| rs7830 | GG 2248 (41.9%) | TG 2349 (43.8%) | TT 768 (14.3%) |

#### Table A3. Unadjusted associations between FeNO and air pollutants PM_10_, NO_2_, NOx, and O_3_ estimated from quantile regression (crude models)

|  |  | FeNO50 (n=4610)  β coefficient (95% CI) | | FeNO270 (n=4283)  β coefficient (95% CI) | | |
| --- | --- | --- | --- | --- | --- | --- |
| Pollutant | Exposure window | 50th percentile | 75th percentile | | 50th percentile | 75th percentile |
| O_3_ | 3 hrs | -0.015  (-0.131, 0.101) | 0.016  (-0.174, 0.206) | | -0.004  (-0.035, 0.027) | 0.013  (-0.039, 0.064) |
|  | 24 hrs | -0.056  (-0.199, 0.086) | -0.032  (-0.267, 0.204) | | 0.012  (-0.027, 0.050) | 0.033  (-0.030, 0.096) |
|  | 120 hrs | -0.117  (-0.302, 0.067) | -0.008  (-0.306, 0.290) | | **0.049**  **(0.001, 0.098)** | 0.075  (-0.004, 0.155) |
| PM_10_ | 3 hrs | -0.075  (-0.265, 0.115) | -0.154  (-0.459, 0.152) | | 0.007  (0.044, 0.057) | 0.033  (-0.051, 0.117) |
|  | 24 hrs | -0.112  (-0.403, 0.180) | 0.088  (-0.384, 0.561) | | 0.011  (-0.066, 0.089) | 0.017  (-0.112, 0.146) |
|  | 120 hrs | -0.158  (-0.535, 0.220) | 0.104  (-0.501, 0.710) | | 0.033  (-0.067, 0.132) | 0.055  (-0.108, 0.218) |
| NO_2_ | 3 hrs | -0.068  (-0.206, 0.070) | -0.098  (-0.328, 0.132) | | -0.002  (-0.039, 0.034) | -0.038  (-0.099, 0.022) |
|  | 24 hrs | -0.113  (-0.357, 0.131) | -0.113  (-0.503, 0.276) | | -0.054  (-0.118, 0.009) | -0.064  (-0.167, 0.039) |
|  | 120 hrs | **-0.350**  **(-0.698, -0.002)** | -0.339  (-0.916, 0.238) | | **-0.156**  **(-0.248, -0.063)** | **-0.194**  **(-0.344, -0.044)** |
| NO_x_ | 3 hrs | 0.000  (-0.039, 0.039) | -0.026  (-0.090, 0.038) | | 0.000  (-0.010, 0.011) | -0.013  (-0.030, 0.004) |
|  | 24 hrs | -0.023  (-0.113, 0.067) | -0.038  (-0.183, 0.107) | | **-0.025**  **(-0.049, -0.001)** | -0.032  (-0.071, 0.007) |
|  | 120 hrs | -0.108  (-0.240, 0.024) | -0.105  (-0.322, 0.111) | | **-0.066**  **(-0.101, -0.031)** | **-0.101**  **(-0.158, -0.044)** |
| Note: Results from single pollutant models. | | | | | | |

#### Table A4. Gene-environment interactions between pollutant exposure and genotypes on FeNO50 estimated from single and multipollutant quantile regression models (50^th^ percentile)

|  | Single pollutant  Marginal effect coefficient (95%CI) | | | | p-value*^a^* | Multi pollutant  Marginal effect coefficient (95%CI) | | | | p-value*^a^* |
| --- | --- | --- | --- | --- | --- | --- | --- | --- | --- | --- |
|  | **rs7830 (NOS3)^b^**  (n=3599) | | | |  | **rs7830 (NOS3)^b^**  (n=3599) | | | |  |
| NO_2_ | GG | | TG/TT | |  | GG | | TG/TT | |  |
| 3 hrs | 0.029  (-0.212, 0.277) | | -0.191  (-0.384, 0.003) | | 0.166 | 0.039  (-0.397, 0.476) | | -0.218  (-0.632, 0.195) | | 0.102 |
| 24 hrs | 0.259  (-0.150, 0.668) | | -0.264  (-0.628, 0.100) | | 0.047 | 0.409  (-0.277, 1.094) | | -0.149  (-0.825, 0.526) | | 0.033 |
| 120 hrs | -0.304  (-0.918, 0.309) | | -0.400  (-0.942, 0.142) | | 0.807 | -0.272  (-1.358, 0.815) | | -0.403  (-1.452, 0.646) | | 0.736 |
|  | | | | | | | | | | |
|  | **rs4795051 (NOS2)^c^**  (n=3612) | | | |  | **rs4795051 (NOS2)^c^**  (n=3612) | | | |  |
| NO_x_ | CC | GC | | GG |  | CC | GC | | GG |  |
| 3 hrs | -0.060  (-0.137, 0.016) | 0.012  (-0.053, 0.076) | | -0.033  (-0.145, 0.080) | 0.155 (GC) | -0.027  (-0.153, 0.098) | 0.073  (-0.043, 0.189) | | 0.034  (-0.111, 0.179) | 0.048 (GC) |
| 24 hrs | -0.174  (-0.387, 0.389) | -0.035  (-0.119, 0.188) | | -0.091  (-0.318, 0.135) | 0.099 (GC) | -0.216  (-0.541, 0.110) | -0.010  (-0.277, 0.257) | | -0.129  (-0.419, 0.161) | 0.103 (GC) |
| 120 hrs | -0.228  (-0.477, 0.020) | -0.086  (-0.335, 0.163) | | -0.086  (-0.448, 0.275) | 0.471 (GC) | -0.115  (-0.501, 0.271) | 0.068  (-0.367, 0.504) | | -0.023  (-0.499, 0.454) | 0.270 (GC) |

Note: All 3-hour interaction analyses are adjusted for age, height, year, month, current cold, and atopy. All 24- and 120-hour interaction analyses are adjusted for age, height, year, month, current cold, atopy, and temperature. All estimates are given per 10 µg/m3 increase in air pollutant exposure.

*^a^* p-value for interaction term. For additive models the genotype with significant difference from the major allele genotype is presented in parentheses.

^b^ Dominant model

^c^ Additive model

#### Table A5. Gene-environment interactions between pollutant exposure and genotypes on FeNO270 estimated from single and multipollutant quantile regression models (GST genes)

| Single pollutant  Marginal effect coefficient (95%CI) | | | p-value*^a^* | Multi pollutant  Marginal effect coefficient (95%CI) | | p-value*^a^* |
| --- | --- | --- | --- | --- | --- | --- |
|  | **rs2266637 (GSTT1)**  (n=2880) | |  | **rs2266637 (GSTT1)**  (n=2880) | |  |
| NO_2_ | CC | GG |  | CC | GG |  |
| 3 hrs | -0.042  (-0.094, 0.010) | -0.002  (-0.107, 0.104) | 0.500 | -0.042  (-0.158, 0.074) | 0.022  (-0.129, 0.172) | 0.275 |
| 24 hrs | -0.016  (-0.110, 0.078) | 0.111  (-0.055, 0.278) | 0.183 | -0.016  (-0.216, 0.184) | 0.118  (-0.120, 0.359) | 0.178 |
| 120 hrs | -0.113  (-0.255, 0.030) | 0.170  (-0.063, 0.403) | 0.038 | -0.035  (-0.314, 0.244) | 0.298  (-0.080, 0.677) | 0.019 |

Note: All 3-hour interaction analyses are adjusted for age, height, year, month, current cold, and atopy. All 24- and 120-hour interaction analyses are adjusted for age, height, year, month, current cold, atopy, and temperature. All estimates are given per 10 µg/m3 increase in air pollutant exposure.

*^a^* p-value for interaction term.

#### Table A6. Gene-environment interactions between pollutant exposure and genotypes on FeNO270 estimated from single and multipollutant quantile regression models (NOS genes)

| Single pollutant  Marginal effect coefficient (95% CI) | | | | | | p-value* | Multi pollutant  Marginal effect coefficient (95% CI) | | | p-value* |
| --- | --- | --- | --- | --- | --- | --- | --- | --- | --- | --- |
|  | **rs4795051 (NOS2)** ^b^  (n=3398) 50^th^ percentile | | | | |  | **rs4795051 (NOS2)** ^b^  (n=3398) 50^th^ percentile | | |  |
| PM_10_ | CC | | GC | | GG |  | CC | GC | GG |  |
| 3 hrs | -0.092  (-0.179,  -0.006) | | 0.073 (0.000, 0.147) | | 0.007  (-0.107, 0.120) | 0.003 (GC) | -0.079  (-0.169, 0.011) | 0.081 (0.004, 0.159) | 0.028  (-0.088, 0.143) | 0.004 (GC) |
| 24 hrs | -0.111  (-0.262, 0.040) | | 0.075  (-0.044, 0.195) | | 0.018  (-0.190, 0.195) | 0.050 (GC) | -0.129  (-0.287, 0.029) | 0.045  (-0.085, 0.175) | -0.004  (-0.218, 0.210) | 0.066 (GC) |
| 120 hrs | -0.031  (-0.222, 0.160) | -0.011  (-0.165, 0.142) | | 0.010  (-0.243, 0.263) | | 0.870 (GC) | -0.031  (-0.236, 0.174) | -0.023  (-0.193, 0.148) | -0.021  (-0.283, 0.241) | 0.944 (GC) |
| NO_2_ |  | | | | |  |  | | |  |
| 3 hrs | -0.084  (-0.147,  -0.020) | -0.009  (-0.063, 0.045) | | -0.001  (-0.098, 0.096) | | 0.160 (GG) | -0.100  (-0.211, 0.012) | -0.033  (-0.140, 0.074) | -0.023  (-0.158, 0.113) | 0.193 (GG) |
| 24 hrs | -0.051  (-0.173, 0.072) | -0.009  (-0.106, 0.088) | | 0.163  (-0.007, 0.333) | | 0.039 (GG) | -0.059  (-0.241, 0.124) | -0.011  (-0.184, 0.162) | 0.157  (-0.075, 0.389) | 0.036 (GG) |
| 120 hrs | -0.188  (-0.359,  -0.018) | -0.007  (-0.150, 0.136) | | 0.234  (-0.008, 0.475) | | 0.004 (GG) | -0.213  (-0.507, 0.082) | -0.049  (-0.308, 0.209) | 0.189  (-0.146, 0.524) | 0.007 (GG) |
| NO_x_ |  | | | | |  |  | | |  |
| 3 hrs | -0.017  (-0.035, 0.001) | 0.003  (-0.012, 0.018) | | 0.001  (-0.025, 0.028) | | 0.250 (GG) | 0.002  (-0.029, 0.032) | 0.019  (-0.009, 0.047) | 0.021  (-0.014, 0.056) | 0.253 (GG) |
| 24 hrs | -0.020  (-0.071, 0.030) | 0.008  (-0.028, 0.045) | | 0.039  (-0.014, 0.093) | | 0.100 (GG) | -0.026  (-0.105, 0.053) | 0.006  (-0.058, 0.071) | 0.047  (-0.023, 0.118) | 0.052 (GG) |
| 120 hrs | -0.057  (-0.115, 0.002) | 0.005  (-0.055, 0.064) | | 0.065  (-0.022, 0.152) | | 0.017 (GG) | -0.014  (-0.106, 0.078) | 0.051  (-0.054, 0.155) | 0.094  (-0.022, 0.209) | 0.035 (GG) |
|  | | | | | | | | | | |
|  | **rs4796017 (NOS2)** ^b^  (n=3378) 75^th^ percentile | | | | |  | **rs4796017 (NOS2)** ^b^  (n=3378) 75^th^ percentile | | |  |
| PM_10_ | AA | GA | | GG | |  | AA | GA | GG |  |
| 3 hrs | -0.038  (-0.182, 0.105) | 0.022  (-0.089, 0.132) | | -0.016  (-0.202, 0.170) | | 0.851 (GG) | -0.045  (-0.203, 0.113) | 0.044  (-0.082, 0.169) | 0.004  (-0.198, 0.207) | 0.693 (GG) |
| 24 hrs | -0.077  (-0.319, 0.165) | -0.075  (-0.261, 0.110) | | 0.181  (-0.139, 0.502) | | 0.199 (GG) | -0.113  (-0.368, 0.143) | -0.091  (-0.293, 0.111) | 0.174  (-0.154, 0.502) | 0.154 (GG) |
| 120 hrs | -0.091  (-0.394, 0.212) | -0.125  (-0.371, 0.121) | | 0.396 (0.003, 0.790) | | 0.047 (GG) | -0.032  (-0.362, 0.297) | -0.040  (-0.318, 0.238) | 0.404  (-0.011, 0.819) | 0.077 (GG) |
|  | | | | | | | | | | |
|  | **rs2248814 (NOS2)** ^b^  (n=3398) 50^th^ percentile | | | | |  | **rs2248814 (NOS2)** ^b^  (n=3398) 50^th^ percentile | | |  |
| PM_10_ | GG | GA | | AA | |  | GG | GA | AA |  |
| 3 hrs | -0.070  (-0.157, 0.017) | 0.055  (-0.023, 0.132) | | 0.078  (-0.041, 0.197) | | 0.033 (GA)  0.046 (AA) | -0.063  (-0.153, 0.027) | 0.052  (-0.028, 0.133) | 0.064  (-0.056, 0.184) | 0.042 (GA)  0.079 (AA) |
| 24 hrs | -0.056  (-0.201, 0.089) | 0.058  (-0.068, 0.184) | | 0.026  (-0.188, 0.240) | | 0.227 (GA)  0.525 (AA) | -0.113  (-0.269, 0.043) | 0.034  (-0.106, 0.175) | 0.039  (-0.186, 0.265) | 0.128 (GA)  0.251 (AA) |
| 120 hrs | 0.044  (-0.143, 0.231) | -0.060  (-0.219, 0.099) | | 0.022  (-0.241, 0.284) | | 0.382 (GA)  0.890 (AA) | 0.039  (-0.161, 0.238) | -0.105  (-0.280, 0.070) | 0.093  (-0.177, 0.364) | 0.219 (GA)  0.728 (AA) |

Note: All 3-hour interaction analyses are adjusted for age, height, year, month, current cold, and atopy. All 24- and 120-hour interaction analyses are adjusted for age, height, year, month, current cold, atopy, and temperature. All estimates are given per 10 µg/m3 increase in air pollutant exposure.

*^a^* p-value for interaction term. For additive models the genotype with significant difference from the major allele genotype is presented in parentheses.

^b^ Additive model

#### Table A7. Gene-environment interactions between pollutant exposure and genotypes on FeNO270 estimated from single and multipollutant quantile regression models (SFTPA genes)

| Single pollutant  Marginal effect coefficient (95% CI) | | | p-value* | Multi pollutant  Marginal effect coefficient (95% CI) | | p-value* |
| --- | --- | --- | --- | --- | --- | --- |
|  | **rs4253527 (SFTPA1)^b^**  (n=3052) | |  | **rs4253527 (SFTPA1)^b^**  (n=3052) | |  |
| O_3_ | CC | TC/TT |  | CC | TC/TT |  |
| 3 hrs | -0.015  (-0.088, 0.058) | 0.155  (0.013, 0.297) | 0.029 | -0.028  (-0.125, 0.069) | 0.1421  (-0.014, 0.299) | 0.027 |
| 24 hrs | -0.022  (-0.128, 0.084) | 0.084  (-0.105, 0.273) | 0.277 | -0.012  (-0.142, 0.119) | 0.095  (-0.109, 0.299) | 0.276 |
| 120 hrs | 0.079  (-0.091, 0.248) | 0.132  (-0.128, 0.393) | 0.671 | 0.078  (-0.118, 0.275) | 0.133  (-0.145, 0.411) | 0.672 |
|  |  | |  |  | |  |
| NO_x_ |  | |  |  | |  |
| 3 hrs | 0.009  (-0.012, 0.029) | -0.037  (-0.078, 0.004) | 0.050 | 0.027  (-0.018, 0.072) | -0.014  (-0.075, 0.046) | 0.089 |
| 24 hrs | 0.021  (-0.033, 0.075) | -0.074  (-0.194, 0.045) | 0.130 | -0.011  (-0.113, 0.091) | -0.092  (-0.238, 0.054) | 0.192 |
| 120 hrs | -0.018  (-0.090, 0.054) | -0.117  (-0.290, 0.055) | 0.269 | -0.022  (-0.171, 0.128) | -0.118  (-0.356, 0.120) | 0.310 |

Note: All 3-hour interaction analyses are adjusted for age, height, year, month, current cold, and atopy. All 24- and 120-hour interaction analyses are adjusted for age, height, year, month, current cold, atopy, and temperature. All estimates are given per 10 µg/m3 increase in air pollutant exposure.

*^a^* p-value for interaction term. For additive models the genotype with significant difference from the major allele genotype is presented in parentheses.

^b^ Dominant model

A B

C

#### Figure A1. SFTPA1 SNPs: Plots of predictive margins with significant interactions. (A) rs4253527 with O_3_ at 3 hours, single pollutant model. (B) rs4253527 with O_3_ at 3 hours, multi pollutant model. (C) rs4253527 with NOx at 3 hours, single pollutant model.

A B

*Figure A2. GSTT1 SNPs: Plots of predictive margins with significant interactions. (A) rs2266637 with NO_2_ at 120 hours, single pollutan tmodel. (B) rs2266637 with NO_2_ at 120 hours, multi pollutant model.*

A B

C

#### Figure A3. NOS2 SNPs: Plots of predictive margins with significant interactions. (A) rs4795051 with PM10 at 3 hours, single pollutant model. (B) rs4795051 with PM10 at 3 hours, multi pollutant model. (C) rs4795051 with PM10 at 24 hours, single pollutant model.

A B

C D

#### Figure A4. NOS2 SNPs: Plots of predictive margins with significant interactions. (A) rs4795051 with NO_2_ at 24 hours, single pollutant model. (B) rs4795051 with NO_2_ at 24 hours, multi pollutant model. (C) rs4795051 with NO_2_ at 120 hours, single pollutant model. (D) rs4795051 with NO_2_ at 120 hours, multi pollutant model.

A

B C

#### Figure A5. NOS2 SNPs: Plots of predictive margins with significant interactions. (A) rs4795051 with NO_x_ at 3 hours, multi pollutant model. (C) rs4795051 with NO_x_ at 120 hours, single pollutant model. (D) rs4795051 with NO_x_ at 120 hours, multi pollutant model.

rs4796017 – PM_10_ at 120 hours

#### Figure A6. NOS2 SNPs: Plots of predictive margins with significant interactions. rs4796017 with PM_10_ at 120 hours, single pollutant model.

A B

#### Figure A7. NOS2 SNPs: Plots of predictive margins with significant interactions. (A) rs2248814 with PM_10_ at 3 hours, single pollutant model. (B) rs2248814 with PM_10_ at 3 hours, multi pollutant model.

A B

#### Figure A8. NOS3 SNPs: Plots of predictive margins with significant interactions. (A) rs7830 with NO_2_ at 24 hours, single pollutant model. (B) rs7830 with NO_2_ at 24 hours, multi pollutant model.
